# Supplementary material for: The journey to diagnosis and care of functional neurological disorder (FND)
Source: PLoS One. 2026 Apr 22;21(4):e0328321. doi: 10.1371/journal.pone.0328321 (PMC13102217; doi:10.1371/journal.pone.0328321)
Supplement: S1 File — (DOCX) [file pone.0328321.s001.docx]

# Interview Schedule: Participants

## **MODIFI: A feasibility study of eye MOvement DesensItisation and reprocessing therapy (EMDR) for FunctIonal neurological disorder (FND)**

I am interested to hear about your experiences of FND, and of being part of the MODIFI trial. I will be asking you some questions to guide the interview. The interview is divided into hearing about your journey from symptoms to diagnosis and any previous treatment; and will then move onto hearing about your experiences of the trial.

(EMDR+NPC only) – As you were allocated to EMDR as part of the trial, I will also be asking about your experiences of EMDR therapy.

1. **FND journey**

To start with I would like to hear a bit about your journey from experiencing symptoms, to diagnosis, to any treatment offered. Please only share what you are happy to talk about.

- Tell me about when you first started experiencing symptoms?
- When they first appeared, what did you think about them?
- Who did you first seek help from?
- When did you first speak to a healthcare professionals about your difficulties?
- Can you remember what that interaction was like for you?
- Which professional diagnosed your symptoms as FND? (e.g. neurologist, GP, neuropsychiatrist)
- Can you recall what that experience was like for you?
- What did you think about the diagnosis initially?
- How long did you wait from starting to experience symptoms to receiving a diagnosis of FND?
- When you attended your neuropsychiatry assessment, what were you hoping for?
- Did you have any ideas regarding what treatment(s) you may be offered?
- Were you hoping for a particular treatment?
- When the neuropsychiatrist mentioned the trial to you, what were your first thoughts?
- Had you heard of EMDR before?
- Other than being referred to the Neuropsychiatry Service, were you referred to any other services?
- Were you offered other treatments?
- If so, did you attend any treatments prior to being seen in the Neuropsychiatry Service?
- If you did attend other treatments, what were they like for you? Did they help?
- What are you hoping for in the future?
